# The exopolysaccharide gene cluster Bcam1330–Bcam1341 is involved in *Burkholderia cenocepacia* biofilm formation, and its expression is regulated by c-di-GMP and Bcam1349

**DOI:** 10.1002/mbo3.61

**Published:** 2012-12-25

**Authors:** Mustafa Fazli, Yvonne McCarthy, Michael Givskov, Robert P Ryan, Tim Tolker-Nielsen

**Affiliations:** 1 Department of International Health, Immunology and Microbiology, Faculty of Health Sciences, University of CopenhagenCopenhagen, Denmark; 2 Department of Microbiology, Biosciences Institute, University College CorkCork, Ireland; 3 Singapore Centre on Environmental Life Sciences Engineering, Nanyang Technological UniversitySingapore

**Keywords:** Biofilm, *Burkholderia cenocepacia*, c-di-GMP, exopolysaccharide

## Abstract

In *Burkholderia cenocepacia,* the second messenger cyclic diguanosine monophosphate (c-di-GMP) has previously been shown to positively regulate biofilm formation and the expression of cellulose and type-I fimbriae genes through binding to the transcriptional regulator Bcam1349. Here, we provide evidence that cellulose and type-I fimbriae are not involved in *B. cenocepacia* biofilm formation in flow chambers, and we identify a novel Bcam1349/c-di-GMP-regulated exopolysaccharide gene cluster which is essential for *B. cenocepacia* biofilm formation. Overproduction of Bcam1349 *in trans* promotes wrinkly colony morphology, pellicle, and biofilm formation in *B. cenocepacia*. A screen for transposon mutants unable to respond to the overproduction of Bcam1349 led to the identification of a 12-gene cluster, Bcam1330–Bcam1341, the products of which appear to be involved in the production of a putative biofilm matrix exopolysaccharide and to be essential for flow-chamber biofilm formation. We demonstrate that Bcam1349 binds to the promoter region of genes in the Bcam1330–Bcam1341 cluster and that this binding is enhanced by the presence of c-di-GMP. Furthermore, we demonstrate that overproduction of both c-di-GMP and Bcam1349 leads to increased transcription of these genes, indicating that c-di-GMP and Bcam1349 functions together in regulating exopolysaccharide production from the Bcam1330–Bcam1341 gene cluster. Our results suggest that the product encoded by the Bcam1330–Bcam1341 gene cluster is a major exopolysaccharide that provides structural stability to the biofilms formed by *B. cenocepacia*, and that its production is regulated by c-di-GMP through binding to and promotion of the activity of the transcriptional regulator Bcam1349.

## Introduction

The *Burkholderia cepacia complex* (*Bcc*) is a group of closely related Gram-negative bacteria containing at least 17 different species. *Bcc* bacteria have recently emerged as highly problematic opportunistic pathogens causing chronic infections in immunocompromised individuals and particularly in patients with cystic fibrosis (CF) (Chiarini et al. 2006). The treatment of patients chronically infected with *Bcc* bacteria is difficult, because these bacteria display high levels of intrinsic antibiotic resistance, persistence in the presence of antimicrobials, and intracellular survival capabilities (reviewed by Chiarini et al. 2006; Drevinek and Mahenthiralingam 2010; Loutet and Valvano 2010). Although all members of *Bcc* have been isolated from CF patients, *Burkholderia cenocepacia* accounts for the majority of these isolates, comprising the most virulent and transmissible strains, often associated with poor clinical course and high mortality in CF patients (Drevinek and Mahenthiralingam 2010). Among the virulence determinants of *B. cenocepacia* identified to date are iron-chelating siderophores, extracellular enzymes, surface polysaccharides and proteins, cell-to-cell signaling, and the ability to form biofilms (Loutet and Valvano 2010).

Biofilms are multicellular communities, in which bacteria are embedded in a self-produced extracellular polymeric matrix, and are often in close association with solid or semisolid surfaces (Costerton et al. 1995). Biofilm bacteria display increased tolerance to antimicrobial treatments and defenses of the host immune system compared with their planktonic counterparts, and they have been implicated in various chronic infectious diseases (Hall-Stoodley and Stoodley 2009; Burmølle et al. 2010). Biofilm formation starts with initial attachment of individual cells to an available surface or to each other. Once irreversibly attached, the bacteria start to proliferate, form microcolonies by clonal growth or aggregation, and develop complex structures (O'Toole and Kolter 1998; Tolker-Nielsen et al. 2000; Stoodley et al. 2002; Klausen et al. 2003a,b). As biofilms mature, the bacteria produce and embed themselves in an extracellular biofilm matrix composed of different types of biopolymers such as exopolysaccharides, proteins, and extracellular DNA (Zogaj et al. 2001; Whitchurch et al. 2002; Friedman and Kolter 2004a,b; Jackson et al. 2004; Matsukawa and Greenberg 2004; Allesen-Holm et al. 2006; Lasa and Penades 2006; Otzen and Nielsen 2008; Nilsson et al. 2011). The biofilm matrix forms a scaffold that holds the biofilm cells together and is responsible for surface adhesion allowing the initial colonization of biotic and abiotic surfaces by planktonic cells, and for the long-term attachment of whole biofilms to surfaces. It also provides the cells with enhanced tolerance to some antibiotics, desiccation, oxidizing agents, and host defenses (reviewed by Pamp et al. 2007 and Flemming and Wingender 2010).

Exopolysaccharides are a major component of the biofilm matrix having roles in attachment and biofilm formation, and they are particularly important for the mechanical stability of biofilms. In many bacteria, including the human pathogens *Escherichia coli*, *Pseudomonas aeruginosa,* and *Vibrio cholerae*, they are indispensable for biofilm formation, and the mutants that cannot produce exopolysaccharides are severely compromised or unable to form mature biofilms (Watnik and Kolter 1999; Danese et al. 2000; Friedman and Kolter 2004a,b; Ryder et al. 2007). Current knowledge shows that *Bcc* bacteria can produce at least four different exopolysaccharides, with the majority of strains producing cepacian (Chiarini et al. 2006), which is thought to be responsible for the mucoid phenotype observed for most of the *Bcc* strains isolated from CF patients (Cescutti et al. 2000; Sist et al. 2003). Analysis of the *B. cenocepacia* J2315 genome revealed that there are several other gene clusters that are implicated in exopolysaccharide biosynthesis (Moreira et al. 2003; Holden et al. 2009), suggesting that the bacterium has the potential to synthesize exopolysaccharides other than the previously identified exopolysaccharides and use them as constituents of its biofilm matrix, probably in response to different stimuli under different environmental conditions.

The intracellular signaling molecule cyclic diguanosine monophosphate (c-di-GMP) plays a central role in the transition between free-living motile and biofilm life styles in many bacteria, and in particular functions as a positive regulator in the synthesis of various biofilm matrix components, including exopolysaccharides (Römling and Simm 2009). The synthesis and degradation of c-di-GMP in bacterial cells are modulated through opposing activities of diguanylate cyclases (DGCs) with GGDEF domain and phosphodiesterases (PDEs) with EAL or HD-GYP domains, respectively (reviewed by Hengge 2009, 2010). We recently showed that GGDEF and EAL domain-mediated c-di-GMP signaling is also operating in *B. cenocepacia* and regulates biofilm formation and virulence (Fazli et al. 2011).

Recently, we provided evidence that Bcam1349, a member of the CRP/FNR family of transcriptional regulators, is a c-di-GMP responsive protein that regulates biofilm formation in *B. cenocepacia* H111, and hypothesized that it does so through regulating the synthesis of extracellular biofilm matrix components (Fazli et al. 2011). Here, we present the results of a genetic screen where we identified a putative exopolysaccharide gene cluster Bcam1330–Bcam1341, expression of which is regulated by c-di-GMP and the Bcam1349 protein, and we demonstrate a role of this gene cluster in *B. cenocepacia* biofilm formation.

## Experimental Procedures

### Strains, plasmids, and growth conditions

The bacterial strains and plasmids used in this study are listed in Table 1. All *B. cenocepacia* and *E. coli* strains were grown at 37°C, and LB medium was used for overnight batch cultivation of the bacterial strains unless otherwise stated. Solid media were solidified with 2% (w/v) agar. Sixty micrograms tetracycline mL^−1^ (liquid medium), 100 μg tetracycline mL^−1^ (solid medium), 25 μg gentamicin-sulfate mL^−1^, and 50 μg kanamycin mL^−1^ were used for the *B. cenocepacia* strains where appropriate. Twenty-five micrograms tetracycline mL^−1^, 25 μg of gentamicin mL^−1^, 50 μg kanamycin mL^−1^, and 6 μg chloramphenicol mL^−1^ were used for the *E. coli* strains where appropriate.

**Table 1:** Bacterial strains and plasmids used in the study

Strain or plasmid	Relevant characteristics	Source or reference
Strains		
* Burkholderia cenocepacia* H111	Clinical isolate from a cystic fibrosis patient	Huber et al. (2001)
* Escherichia coli* SM10-λpir	Host of the pBT20 Tn-mariner delivery vector	Herrero et al. (1990)
* E. coli* S17-1-λpir	Host of the mini-Tn7-kan-*gfp* delivery vector	Simon et al. (1982)
* E. coli* DH5α	Used for standard DNA manipulations	Invitrogen
* E. coli* DB3.1	Host for the gene replacement vectors pEX18AP-*pheS* and pEX18Ap-*pheS*-GW	Invitrogen
Plasmids		
pBBR1MCS2	Broad-host range cloning vector, Km^R^	Kovach et al. (1995)
pBcam1349	pBBR1MCS2 with *bcam1349* inserted in BamHI/XbaI sites	Fazli et al. (2011)
pRK404A	Broad-host range cloning vector, Tet^R^	Ditta et al. (1985)
pYedQ (pYhcK)	pRK404A with the *E. coli yedQ* (yhcK) gene inserted	Ausmees et al. (2001)
pBT20	Tn mariner delivery vector, Gm^R^	Kulasekara et al. (2005)
pmini-Tn7-kan-*gfp*	Delivery vector for mini-Tn7-kan-*gfp*, Km^R^	Norris et al. (2010)
pUX-BF13	mob^+^ ori-R6K; helper plasmid providing theTn7 transposition functions in trans. Amp^R^	Bao et al. (1991)
pRK600	Helper plasmid in matings, Cm^R^, ori-ColE1 RK-mob+ RK2-tra+	Kessler et al. (1992)
pEX18AP-*pheS*	Gene replacement vector based on *pheS* and Ap^R^	Barrett et al. (2008)
pEX18ApGW	Gateway compatible gene replacement vector, Suc^S^Ap^R^, Cm^R^	Choi and Schweizer (2005)
pEX18Ap-*pheS*-GW	Gateway cassette (1.74-kb *Kpn*I/*Sph*I fragment) from pEX18ApGW cloned in *Kpn*I/*Sph*I digested pEX18Ap-*pheS*	This study
pDONR221	Gateway donor vector, Km^R^	Invitrogen
pPS856	0.83-kb blunt-ended SacI fragment from pUCGM ligated into the EcoRV site of pPS854. Ap^R^, Gm^R^	Hoang et al. (1998)
pEX18Ap-*pheS*-GW- *bcal1389*	Gene replacement vector for *bcal1389*. Ap^R^, Gm^R^	This study
pEX18Ap-*pheS*-GW- *bcal1677*	Gene replacement vector for *bcal1677*. Ap^R^, Gm^R^	This study

### Transposon mutagenesis and screening for mutants

Transposon mutagenesis was performed by use of the Tn-Mariner transposon vector pBT20 (Kulasekara et al. 2005). The donor strain (*E. coli* SM 10-λpir/pBT20) and the recipient strain (*B. cenocepacia* carrying a plasmid-borne *bcam1349*) were harvested from overnight culture on LB-agar plates containing appropriate antibiotics, and were resuspended in 1 mL 0.9% NaCl. Concentrations of the cells in the suspensions were adjusted to an OD_600_ of 40 and 20 for the donor and the recipient strain, respectively. Fifty microliters of each strain were mixed, and spot inoculated on prewarmed LB-agar plates. After 45 min of incubation at 37°C, the cells were scraped off and were resuspended in 1 mL of 0.9% NaCl. The cell suspension was plated on AB-agar medium (without NaCl) supplemented with 10 mmol/L Na-citrate as carbon source and 25 μg gentamicin sulfate mL^−1^. The plates were incubated at 37°C for 4 days. Thirteen independent rounds of transposon mutagenesis were performed as described above, and approximately 6000 colonies were screened for smooth colony morphology. To determine the location of the transposon insertions, arbitrary primed PCR was performed with genomic DNA isolated from the mutants (O'Toole et al. 1999), and the sequence of the PCR products was compared with the *B. cenocepacia* J2315 genome at *Burkholderia* Genome Database.

### Construction of *bcal1389* and *bcal1677* deletion mutants

Gene replacement fragments containing a gentamicin (Gm) resistance cassette were generated by PCR overlap extension as described by Choi and Schweizer (2005). Primers were used to amplify chromosomal regions upstream and downstream of Bcam1389 and Bcal1677 and to amplify a Gm resistance cassette from plasmid pPS856 (Hoang et al. 1998). The PCR fragments were fused together and amplified with primers GW-attB1 and GW-attB2 incorporating the *att*B1 and *att*B2 recombination sites at either end of the gene replacement cassette. Using the Gateway cloning system (Invitrogen, Life Technologies, Denmark), the resulting gene replacement fragments were first transferred into pDONR221 generating entry plasmids, and subsequently transferred into pEX18Ap-*pheS*-GW generating the gene replacement plasmids pEX18AP-*pheS*-GW-*bcal1389* and pEX18AP-*pheS*-GW-*bcal677*.

To construct the gene replacement plasmid pEX18Ap-*pheS*-GW used in this study, a 1744-bp GW cassette was excised from pEX18ApGW (Choi and Schweizer 2005) by restriction with *Kpn*I/*Sph*I and was inserted into the *Kpn*I/*Sph*I digested pEX18Ap-*pheS* (Barrett et al. 2008). The presence of the insertion was confirmed by restriction analysis.

To construct the *bcal1389* and *bcal1677* deletion mutants, the gene replacement plasmids pEX18AP-*pheS*-GW-*bcal1389* and pEX18AP-*pheS*-GW-*bcal677* were transferred into *B. cenocepacia* H111 by tri-parental mating as described previously (Fazli et al. 2011), and the resulting transformants were selected on AB-agar plates supplemented with 10 mmol/L Na-citrate and 25 μg gentamicin sulfate mL^−1^. Resolution of single crossover events was achieved by streaking on plates containing 0.1% (w/v) *p*-chlorophenylalanine (cPhe; DL-4-chlorophenylalanine; Sigma-Aldrich, Brøndby, Denmark) via the counter-selectable *pheS* marker on the gene replacement plasmid (Barrett et al. 2008).

### Colony morphology assay

Colony morphology of the *B. cenocepacia* strains was investigated by spot-inoculating 3 μL of overnight-grown cultures on AB-agar medium (AB medium containing 0.2% [w/v] glucose, and solidified with 2% [w/v] agar) without NaCl, and subsequent incubation at 37°C for 2 days.

### Pellicle assay

Overnight cultures were 100 times diluted in 5 mL of LB medium, supplemented with appropriate antibiotics for maintenance of the plasmids, and were subsequently placed in culture tubes. Pellicles were assayed by visual inspection of the air–liquid interface of the static liquid culture after 2 days at room temperature. Complete coverage of the surface with an opaque layer of cells and matrix material was considered pellicle formation.

### *gfp*-tagging of strains and cultivation of flow-cell biofilms

In order to tag the *B. cenocepacia* strains with *gfp*, the strain containing the delivery plasmid pmini-Tn7-kan-gfp, the strains containing the helper plasmids pUX-BF13 and pRK600, and the recipient strain were grown in LB medium with appropriate antibiotics at 37°C overnight. Five hundred microlitres of the overnight-grown strains containing the delivery and helper plasmids, and 250 μL of the recipient strain were mixed, washed twice in 1 mL LB medium, and resuspended in 100 μL LB. The cell suspension was spot inoculated on prewarmed LB-agar plates and incubated 37°C overnight. The next day, the cells were scraped from the plates and resuspended in 1 mL 0.9% NaCl. Serial dilutions of the cell suspension were plated on selective AB-agar medium supplemented with 10 mmol/L Na-citrate as carbon source and 50 μg kanamycin mL^−1^, and incubated at 37°C for colony growth. The transformants were checked whether they were tagged with *gfp* by using an epifluorescence microscope equipped with filters to detect *gfp*-fluorescence.

Biofilms were grown at 37°C in three-channel flow chambers with channel dimensions of 1 × 4 × 40 mm irrigated with AB minimal medium (Pamp and Tolker-Nielsen 2007) supplemented with trace metals (200 μL L^−1^) and glucose (1 mmol/L) as carbon source. The flow-chamber biofilm system was assembled and prepared as described previously (Crusz et al. 2012). The substratum consisted of a 24 × 50 mm microscope glass cover slip. Overnight cultures of the relevant strains were diluted 100 times in 0.9% NaCl. Three hundred microliters of the diluted bacterial cultures were inoculated by injection into the flow chambers. After inoculation, the flow chambers were allowed to stand inverted, with no flow, for 1 h. Medium flow was resumed with flow chambers standing upright. A peristaltic pump (Watson-Marlow 250S, Ringsted, Denmark) was used to keep the medium flow at a constant rate of 3 mL h^−1^. Flow-chamber biofilm experiments were carried out at least three times with two replicates in each experiment.

### Microscopy and image analysis

Two flow-cell channels per strain were inoculated and biofilm formation was monitored on a daily basis for 3 days (∼72 h). Microscopic observation and image acquisition of biofilms were performed with a Zeiss LSM 710 CLSM (Carl Zeiss Microscopy, Jena, Germany) equipped with an argon laser and detectors and filter sets for monitoring *gfp*-fluorescence. Images were obtained using a 63×/1.4 objective. Simulated fluorescence projections were generated using the imaris software package (Bitplane AG, Zurich, Switzerland). Images were further processed for display by using Photoshop software (Adobe, Copenhagen, Denmark). Quantification of biofilm biomass was performed by COMSTAT software (Heydorn et al. 2000). For COMSTAT analysis, a total of 12 image stacks (six image stacks from each flow-cell channel) were acquired randomly for each strain from flow-cell biofilm experiments.

### LPS profiling by SDS-PAGE

Lipopolysaccharide was extracted from bacteria as described previously (Nilsson et al. 2011). Briefly, 10–15 colonies grown on AB glucose-agar medium without NaCl at 37°C for 2 days were suspended in 100 μL lysis buffer containing 10 mmol/L Tris–HCl (pH 8.0), 2% SDS, incubated at 100°C for 10 min. LPS was extracted with equal volume of 90% phenol at 70°C for 12 min and subsequent incubation in ice bath for 2 min. The suspension was centrifuged at 1000 *g* for 10 min, and the aqueous phase was transferred to a new microcentrifuge tube. The LPS extract was separated on a 15% SDS-PAGE gel, and visualized using the Bio-Rad silver stain kit according to manufacturer's instructions (Bio-Rad, Copenhagen, Denmark).

### Quantitative real-time PCR assays

The effect of increased cellular levels of Bcam1349 and c-di-GMP on expression of the genes of the Bcam1330–Bcam1341 cluster was monitored by quantitative RT-PCR. RNA was isolated from biofilm grown *B. cenocepacia* wild type with and without the plasmid pBcam1349 or pYedQ and their corresponding vector control strains on membrane filters placed on AB glucose-agar medium for 48 h using the RNeasy Protect Bacteria Mini Kit (Qiagen, Copenhagen, Denmark), and it was DNase treated using the Turbo DNA-free kit (Ambion, Life Technologies, Denmark) according to manufacturers' instructions. cDNA synthesis and quantitative RT-PCR analysis were carried out using the Qscript 1-Step Sybr green qRT-PCR kit (Quanta Biosciences, Gaithersburg, MD) according to manufacturer's instructions. As a control, quantitative RT-PCR was similarly applied to analyze the expression of the *gyrB* gene. The relative expression levels of the target genes were calculated using the threshold cycle (2^−ΔΔCt^) method (Livak and Schmittgen 2001).

### 5′ RACE

The Invitrogen 5′ RACE System (version 2.0) was used to determine the transcription start site (TSS) of the *bcam1330* and *bcam1331* genes according to manufacturer's instructions using the Abridged Anchor Primer (AAP) and Abridged Universal Amplification Primer (AUAP) in combination with the gene-specific primers. Total RNA was isolated from *B. cenocepacia* cultures grown until midexponential phase using the RNeasy Protect Bacteria Mini Kit (Qiagen).

### Electrophoretic mobility shift assays

The DNA probes containing the putative promoter regions (500 basepairs) of the genes of interest were generated by PCR using 5′-end DIG synthetic oligonucleotides as primers. Isolation of the Bcam1349 protein, the binding conditions, and the detection procedures were as described previously (Fazli et al. 2011).

## Results

### Cellulose and type-I fimbriae genes are not essential for *B. cenocepacia* biofilm formation

In a previous study, we provided evidence that the transcriptional regulator Bcam1349 is a c-di-GMP responsive protein that regulates biofilm formation in *B. cenocepacia* (Fazli et al. 2011). We found by comparative proteomics analysis that expression of a cellulose biosynthesis protein (Bcal1391) and a fimbrial protein (Bcal1677) were downregulated in the *bcam1349* mutant, and subsequently showed by quantitative real-time polymerase chain reaction (qRT-PCR) that the transcript levels of all the cellulose biosynthesis genes were significantly decreased in the *bcam1349* mutant compared with the wild type. Furthermore, we showed that the Bcam1349 protein binds to *bcs* (bacterial cellulose synthase) promoter DNA in a manner that is promoted by c-di-GMP. Based on these results, we hypothesized that a decrease in cellulose and fimbriae expression in the *bcam1349* mutant may be an underlying cause of the biofilm defect displayed by this mutant.

In this study, we generated *bcal1389* and *bcal1677* mutants by replacing the corresponding open reading frames (ORFs) with a gentamicin resistance cassette. We chose to delete these genes, because *bcal1389* and *bcal1677* are the first genes of the putative cellulose (Bcal1389–Bcal1397) and fimbriae (Bcal1677–Bcal1680) biosynthesis gene clusters, respectively. Analysis of the two gene clusters by using the DOOR (Database of prokaryotic operons) operon prediction tool (Dam et al. 2007; Mao et al. 2009) predicted that the genes of the cellulose biosynthesis cluster form two operons, Bcal1389–Bcal1390 and Bcal1391–Bcal1397, while the genes of the fimbriae biosynthesis cluster form a single operon. Thus, replacement of the *bcal1389* and *bcal1677* genes with the gentamicin resistance cassette is expected also to prevent expression of the downstream genes in the operons due to the presence of a transcriptional terminator in the gentamicin resistance cassette.

**Figure 1:**
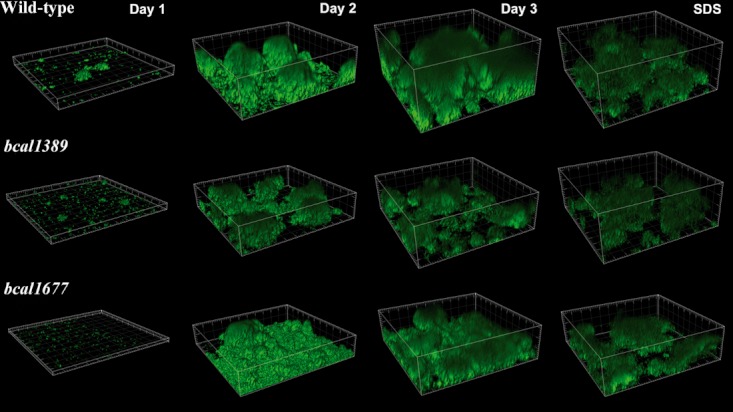
Confocal laser scanning microscope (CLSM) images of flow-cell grown *Burkholderia cenocepacia* wild type and mutant biofilms were acquired on a daily basis for 3 days. On day 3, CLSM images were acquired before and after sodium dodecyl sulfate treatment. The dimension of each image is 220 × 220 μm.

**Figure 2:**
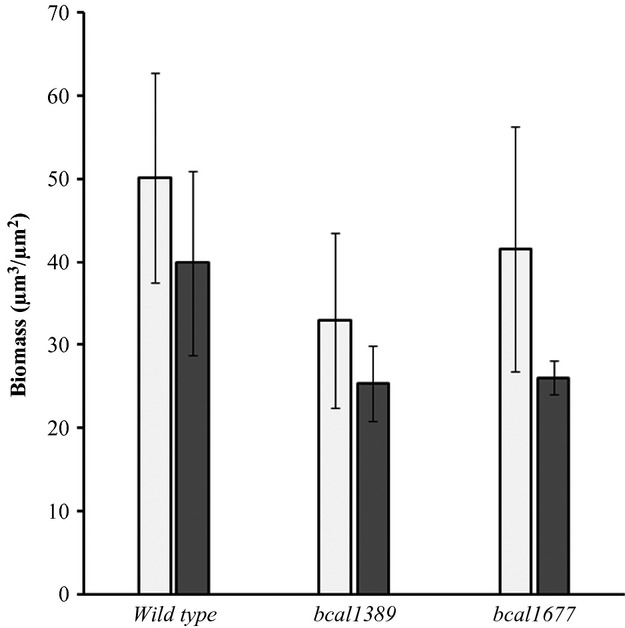
Quantitative analysis of the amount of biofilm formed in flow-cells by *Burkholderia cenocepacia* wild type and mutant strains. Twelve confocal laser scanning microscope (CLSM) image stacks were acquired at random positions in 3-day-old biofilms before and after sodium dodecyl sulfate (SDS) treatment, and the biomass was quantified by COMSTAT analysis. White columns, before SDS treatment; gray columns, after SDS treatment. The error bars represent the standard deviation between 12 CLSM image stacks.

To elucidate whether the cellulose and fimbrial biosynthesis proteins are required for biofilm formation in *B. cenocepacia* H111, we investigated the biofilm formation ability of the *bcal1389* and *bcal1677* mutants in a flow-cell biofilm system irrigated with glucose medium. The mutant strains formed biofilms similar to the wild-type biofilm with highly structured large microcolonies (Fig. 1). However, confocal laser scanning microscope (CLSM) micrographs of the biofilms formed by the mutants appeared less bright than the corresponding CLSM micrographs of the wild-type biofilm, indicating that there is a lower cell density in the mutant biofilms than in the wild-type biofilm (Fig. 1). This less dense biofilm structure observed for the mutants is reflected in the results obtained by COMSTAT analysis, where there is a reduction of the biomass in the mutant biofilms compared with the wild-type biofilm (Fig. 2). The less dense structure of the mutant biofilms could be due to a lack of cell-to-cell connecting cellulose or fimbriae, making the cells less cohesive to each other. To evaluate whether the stability of the *bcal1389* and *bcal1677* mutant biofilms was impaired, we treated 3-day-old flow-cell biofilms with sodium dodecyl sulfate (SDS). Treatment with medium containing 0.03% SDS for 1 h caused almost the same reduction of the biomass in the wild-type and mutant biofilms, indicating that the mutant biofilms were nearly as resistant to SDS treatment as the wild-type biofilm, and that cellulose and fimbriae do not play a major role in biofilm stability under the conditions tested (Figs. 1, 2).

**Figure 3:**
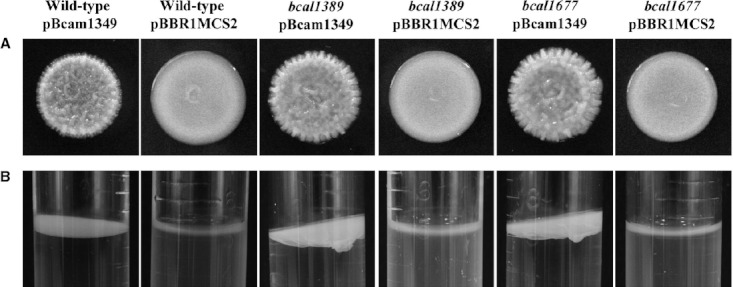
Colony morphology on AB-agar medium (A), and pellicle formation in static LB liquid culture (B) of the wild type, and *bcal1389* and *bcal1677* mutant strains carrying either pBcam1349 or pBBR1MCS2 (vector control).

When we overexpress the *bcam1349* gene from the multicopy number plasmid pBcam1349 in wild-type *B. cenocepacia*, the bacteria form wrinkled colonies on AB-agar medium (Fig. 3A) and thick pellicles at the air–liquid interface of standing cultures, unlike the corresponding vector control strain (Fig. 3B). These altered phenotypes were also observed in wild-type *B. cenocepacia* as a response to elevated intracellular c-di-GMP levels (Fazli et al. 2011). We previously showed that overproduction of Bcam1349 in the cell does not alter the intracellular c-di-GMP levels (Fazli et al. 2011). Therefore, the formation by the pBcam1349-containing bacteria of wrinkled colonies and thick pellicles suggested that expression of *bcam1349* from the pBcam1349 plasmid leads to excessive amounts of Bcam1349 protein, which then may bind c-di-GMP from the native cellular pool and stimulate gene expression. If the above-mentioned phenotypic changes are caused by upregulation of the cellulose and fimbriae biosynthesis genes through overproduction of the Bcam1349 protein in the wild type, then mutations in the *bcal1389* and *bcal1677* genes should reverse these phenotypes. To test this hypothesis, we overexpressed *bcam1349* from pBcam1349 in the *bcal1389* and *bcal1677* mutant strains. The mutants overproducing the Bcam1349 protein formed wrinkled colonies on agar medium and thick pellicles in standing cultures like the wild-type/pBcam1349 strain, while the vector control strains formed smooth colonies and failed to form pellicles (Fig. 3A and B), suggesting that the cellulose and fimbriae genes are not involved in wrinkled colony morphology and increased pellicle formation in the wild type/pBcam1349 strain.

Taken together, our results with the *bcal1389* and *bcal1677* mutants suggest that cellulose and type-I fimbriae are not important for biofilm formation and biofilm stability in *B. cenocepacia* under the conditions tested, and that Bcam1349 regulates expression of other genes encoding products that are possibly more important for biofilm formation and biofilm stability than cellulose and type-I fimbriae.

### Isolation of mutants defective in forming Bcam1349-induced wrinkled colony morphology

**Figure 4:**
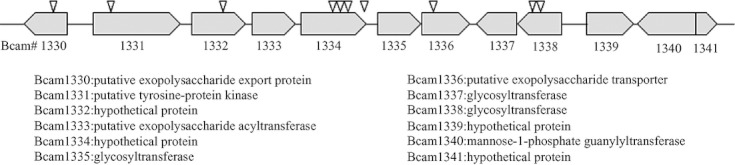
The putative exopolysaccharide biosynthesis gene cluster Bcam1330–Bcam1341 from the sequenced genome of *Burkholderia cenocepacia* J2315. The putative genes are drawn to scale, and transposon insertions are indicated by inverted triangles. The annotated protein functions are written below.

Wrinkled colony morphology on solid medium depends on the ability of bacteria to self-organize and produce biofilm matrix components such as exopolysaccharides, and it has been found highly correlated with increased biofilm formation ability of various bacteria (Rainey and Travisano 1998; Spiers et al. 2002, 2003; Friedman and Kolter 2004a,b; Branda et al. 2005). In this study, formation of wrinkled colonies by the pBcam1349-containing wild-type strain (Fig. 3A) provided a basis for identification of biofilm matrix genes whose expression is potentially regulated by the Bcam1349 protein in *B. cenocepacia*. We performed transposon mutagenesis in the pBcam1349-containing wild-type strain and screened approximately 6000 transposon insertion mutants for smooth colony morphology on AB-agar medium. The mutants that gave rise to smooth colony morphology were isolated and characterized further. To determine the location of the transposon insertions, the DNA region flanking the transposon insertion site in the mutants was sequenced and compared with the *B. cenocepacia* J2315 genome by using the BLASTN tool of the Burkholderia Genome Database. Ten mutants were found to have the transposon insertion in six genes of a 12-gene cluster (Bcam1330–Bcam1341) predicted to be involved in exopolysaccharide biosynthesis (Fig. 4).

### Sequence analysis of the Bcam1330–Bcam1341 exopolysaccharide biosynthesis gene cluster

The Bcam1330–Bcam1341 gene cluster is one of the putative exopolysaccharide biosynthetic clusters identified in the *B. cenocepacia* J2315 genome (Holden et al. 2009), spanning a region of 18.5 kb with 12 ORFs, eight in the plus strand and four in the minus strand. Most of the ORFs are separated by small intergenic regions or show small overlap with the adjacent gene (Fig. 4). Analysis of the Bcam1330–Bcam1341 gene cluster by using DOOR (Dam et al. 2007; Mao et al. 2009) revealed that six genes of the cluster are present as three different operons: Bcam1335–1336, Bcam1337–1338, and Bcam1340–1341. The genes in the cluster encode proteins with putative exopolysaccharide-related functions (Table 2).

**Table 2:** Bioinformatic analysis of the Bcam1330–Bcam1341 gene cluster

Gene no	Product name	COG/TIGRFAM/pfam no., description	Predicted domains	Subcellular localization
Bcam1330	Putative exopolysaccharide export protein	COG1596, Wza, periplasmic protein involved in polysaccharide export; pfam02563, polysaccharide biosynthesis/export protein	1 transmembrane; Poly_export; SLBB	Outer membrane
Bcam1331	Putative tyrosine-protein kinase	COG3206, GumC, uncharacterized protein involved in exopolysaccharide biosynthesis; pfam02706, Wzz, chain length determinant protein	2 transmembrane; Wzz; CbiA nucleotide binding	Cytoplasmic membrane
Bcam1332	Hypothetical protein	–	12 transmembrane	Cytoplasmic membrane
Bcam1333	Putative exopolysaccharide acyltransferase	COG1835, predicted acyltransferase; pfam01757, acyltransferase family	10 transmembrane; Acyl_transf_3	Cytoplasmic membrane
Bcam1334	Hypothetical protein	TIGR03100, predicted hydrolase of the alpha/beta superfamily (TIGRFAM prediction)	–	Unknown
Bcam1335	Glycosyltransferase	COG0438, RfaG, Glycosyltransferase; pfam00534, glycosyltransferase group 1 family protein	Glycos_transf_1	Cytoplasmic
Bcam1336	Putative exopolysaccharide transporter	COG2244, RfbX, membrane protein involved in the export of O-antigen and teichoic acid; pfam01943, polysaccharide biosynthesis protein	10 transmembrane; Polysacc_synt	Cytoplasmic membrane
Bcam1337	Glycosyltransferase	COG0438, RfaG, glycosyltransferase; pfam00534, glycosyltransferase group 1 family protein	Glycos_transf_1	Cytoplasmic
Bcam1338	Glycosyltransferase	COG0438, RfaG, glycosyltransferase; pfam00534, glycosyltransferase group 1 family protein	Glycos_transf_1	Unknown
Bcam1339	Hypothetical protein	TIGR03805, parallel beta-helix repeat-containing protein (TIGRFAM prediction)	1 transmembrane; eight PbH1, parallel beta-helix repeats	Unknown
Bcam1340	Mannose-1-phosphate guanylyltransferase	COG0836, ManC, mannose-1-phosphate guanylyltransferase; pfam00483, nucleotidyltransferase	NTP_transferase; MannoseP_isomer; Cupin_2	Cytoplasmic
Bcam1341	Hypothetical protein	COG2153, predicted acyltransferase	–	Cytoplasmic

### Phenotypic characterization of mutants suggests that the bcam1330–bcam1341 gene cluster encodes an exopolysaccharide essential for *B. cenocepacia* biofilm formation

**Figure 5:**
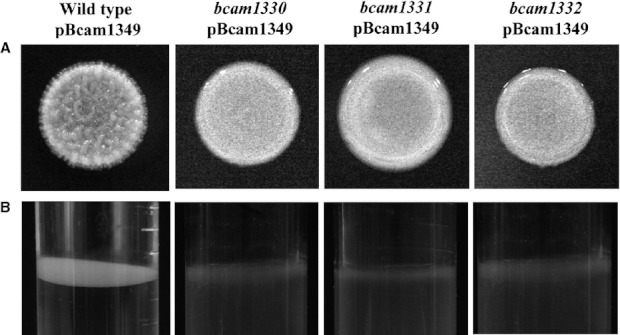
Colony morphology on AB-agar medium (A), and pellicle formation in static LB liquid culture (B) of the transposon mutants containing the plasmid pBcam1349.

We chose to characterize the *bcam1330*, *bcam1331,* and *bcam1332* mutants further in this study. The sequence analysis of the Bcam1330–Bcam1341 gene cluster led us to hypothesize that it is involved in the production of an exopolysaccharide, and our observations with the mutants support this hypothesis. The colonies formed by the pBcam1349-containing wild type had wrinkled colony morphology, whereas those formed by the pBcam1349-containing *bcam1330*, *bcam1331,* and *bcam1332* mutants were smooth (Fig. 5A). In addition, the cells in the pBcam1349-containing wild-type colonies seemed to be encased in a matrix material as the colonies could easily be removed as one piece from the agar plate, whereas this was not the case for pBcam1349-containing mutants (data not shown). Furthermore, unlike the pBcam1349-containing wild type, the pBcam1349-containing mutants were unable to form pellicles at the air–liquid interface of static cultures (Fig. 5B). Wrinkled colony morphology in *V. cholerae*, *Salmonella Typhimurium,* and *Pseudomonas fluorescens* and pellicle formation in *P. aeruginosa* have previously been associated with the ability of the bacteria to produce exopolysaccharides (Yildiz and Schoolnik 1999; Zogaj et al. 2001; Spiers et al. 2002). Thus, the smooth colony morphology and loss of pellicle formation in the mutants are consistent with an inability to produce one or more biofilm matrix exopolysaccharides, further supporting our hypothesis that the Bcam1330–Bcam1341 gene cluster is involved in the production of a biofilm matrix exopolysaccharide.

Because expression of exopolysaccharide genes in some cases affects regulation of lipopolysaccharide (LPS) production or results in gene products that constitute a portion of LPS biosynthesis pathways, the defect in biofilm formation by the *bcam1330*, *bcam1331,* and *bcam1332* mutants could potentially be due to defects in LPS production instead of defects in exopolysaccharide production, as LPS previously has been implicated in *P. aeruginosa* and *P. fluorescens* biofilm formation (Rocchetta et al. 1999; Spiers and Rainey 2005). To test this hypothesis, we extracted LPS from the *bcam1330*, *bcam1331,* and *bcam1332* mutants that were cured from the plasmid pBcam1349 along with the wild type strain, resolved it by SDS-PAGE and visualized it by silver staining. The mutants showed an LPS profile similar to that of the wild type, suggesting that the mutants are not affected in LPS production (data not shown).

**Figure 6:**
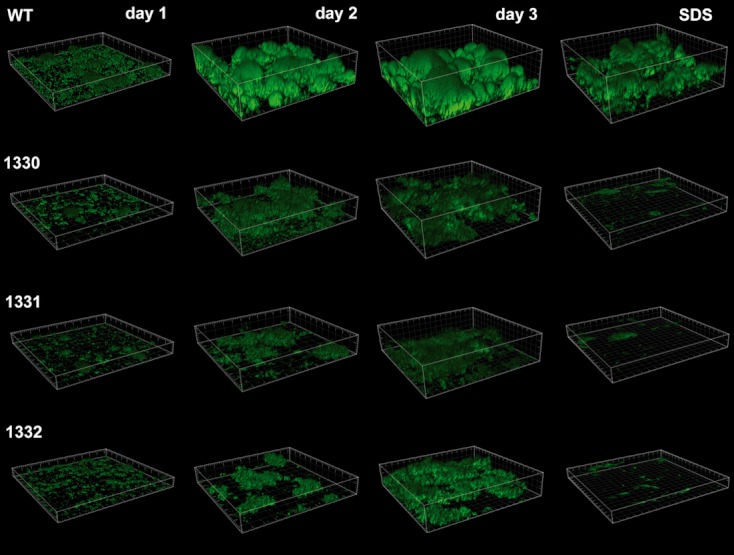
Confocal laser scanning microscope (CLSM) images of flow-cell grown *Burkholderia cenocepacia* wild type and transposon mutant biofilms were acquired on a daily basis for 3 days. On day 3, CLSM images were acquired before and after sodium dodecyl sulfate treatment. The dimension of each image is 220 × 220 μm.

**Figure 7:**
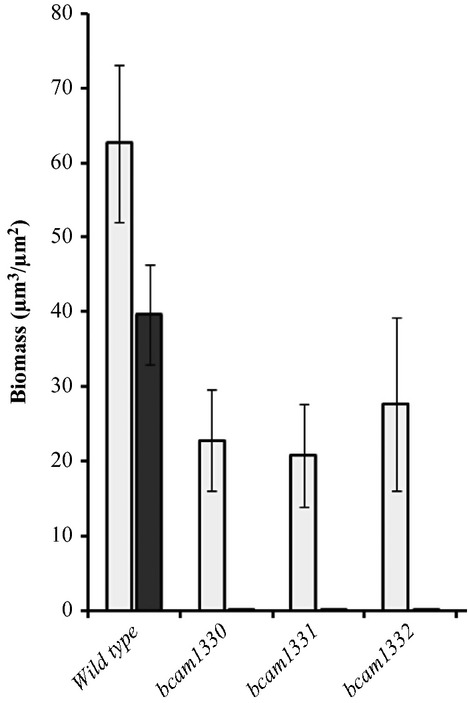
Quantitative analysis of the amount of biofilm formed in flow-cells by *Burkholderia cenocepacia* wild type and transposon mutant strains. Twelve confocal laser scanning microscope (CLSM) image stacks were acquired at random positions in 3-day-old biofilms before and after sodium dodecyl sulfate (SDS) treatment, and the biomass was quantified by COMSTAT analysis. White columns, before SDS treatment; gray columns, after SDS treatment. The error bars represent the standard deviation between 12 CLSM image stacks.

Exopolysaccharides comprise a major component of the biofilm matrix and contribute to attachment, biofilm formation, and biofilm stability in many bacteria, including the human pathogens *E. coli*, *P. aeruginosa,* and *V. cholerae* (Watnik and Kolter 1999; Danese et al. 2000; Ryder et al. 2007; Colvin et al. 2011). Since we hypothesized that the Bcam1330–Bcam1341 gene cluster is involved in the production of a putative biofilm matrix exopolysaccharide, we found it of interest to investigate biofilm formation by the mutants in our flow-cell biofilm system. First, we cured the mutants from the pBcam1349 plasmid by growing them in serial passages in medium without the antibiotic required for maintenance of the plasmid. As shown in Figure 6, the mutants did not seem to be severely affected in initial surface attachment as they were able to colonize the glass surface and form small microcolonies after 1 day of cultivation. However, the mutants displayed dramatic defects in the later stages of biofilm development. They formed biofilms with irregular and protruding structures floating in the direction of medium flow in contrast to the wild type, which formed highly structured robust biofilms in the flow cells (Fig. 6), and they produced substantially reduced levels of biofilm biomass compared with the wild type (Fig. 7). Furthermore, cell aggregates were detaching from these protruding biofilm structures at an increased rate, which indicated that the mutant biofilms lack mechanical stability leading to reduced resistance against the shear stress caused by the medium flow. When treated with medium containing 0.03% SDS for 1 h, the mutant biofilms were completely washed away while the wild-type biofilm was resistant to SDS treatment (Figs. 6, 7), providing additional evidence that the mutant biofilms lack mechanical stability due to the loss of an extracellular biofilm matrix component.

**Figure 8:**
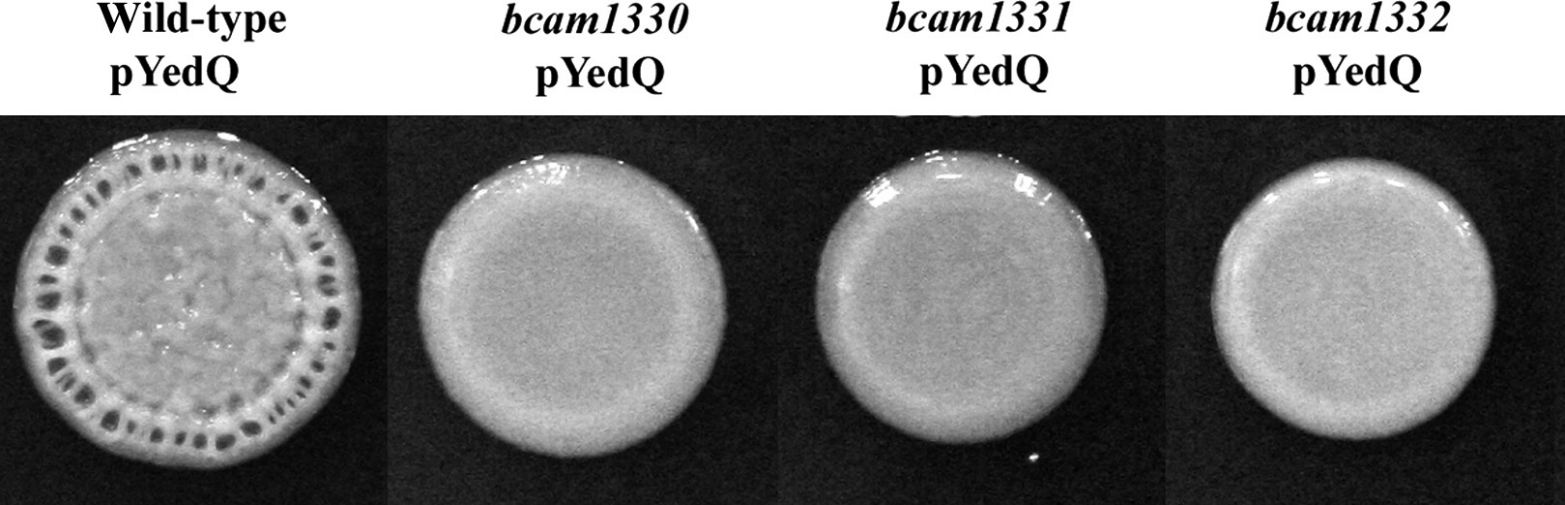
Colony morphology on AB-agar medium of the wild type and transposon mutants with plasmids pYedQ.

If expression of the Bcam1330–Bcam1341 gene cluster is regulated by Bcam1349 in a manner that is promoted by high intracellular levels of c-di-GMP (i.e., if Bcam1349 and c-di-GMP influence gene expression at the same point), and if the product encoded by the Bcam1330–Bcam1341 gene cluster is the major contributor to the wrinkly colony morphology observed either when Bcam1349 or c-di-GMP is overproduced, then an increase in the level of c-di-GMP should not lead to the formation of wrinkled colony morphology in the *bcam1330*, *bcam1331,* and *bcam1332* mutants. To test this hypothesis, we introduced the *yedQ* containing plasmid pYedQ into the cured mutants and observed the phenotypic changes. The pYedQ plasmid harbors the DGC gene *yedQ,* expression of which has previously been shown to greatly elevate the level of c-di-GMP in *B. cenocepacia* (Fazli et al. 2011). The *bcam1330*, *bcam1331,* and *bcam1332* mutants containing the pYedQ plasmid did not form wrinkled colonies on agar medium unlike the wild type/pYedQ strain (Fig. 8), supporting our hypothesis that c-di-GMP and Bcam1349 function in the same pathway, regulating production of the putative biofilm matrix exopolysaccharide through modulating the expression of one or more genes in the Bcam1330–Bcam1341 gene cluster.

As multiple different transposon insertions occurred in the Bcam1330–Bcam1341 gene cluster and they all resulted in similar phenotypes, we excluded the possibility that the observed phenotypes were due to secondary mutations. Thus, we did not carry out complementation analysis for the *bcam1330*, *bcam1331,* and *bcam1332* mutants.

### Expression of the *bcam1330* and *bcam1331* genes is increased in strains with high levels of c-di-GMP or Bcam1349

**Figure 9:**
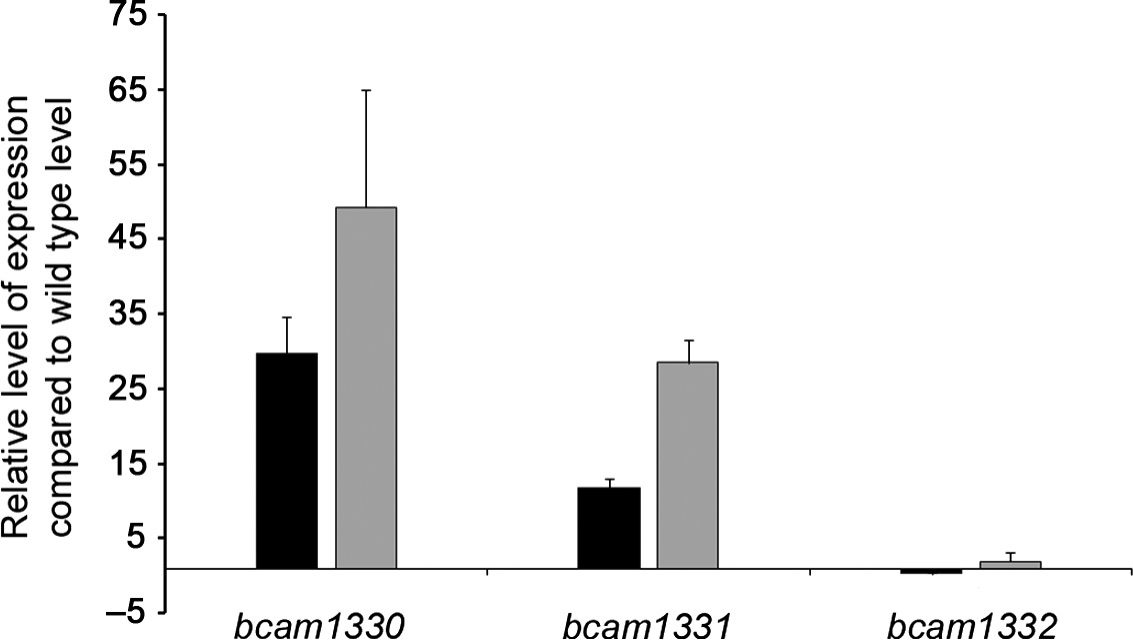
qRT-PCR analysis of transcript levels of the *bcam1330*, *bcam1331*, and *bcam1332* genes in the *Burkholderia cenocepacia* wild type vector control strains, Bcam1349-overproducing wild type/pBcam1349 strain (black columns) and the wild-type/pYedQ strain with elevated intracellular levels of cyclic diguanosine monophosphate (gray columns). Data are normalized to *gyrB* transcript levels and presented as the fold change with respect to the wild type for each gene. Data (means ± SD) are representative of three independent biological experiments.

Based on the results so far, we suggest that c-di-GMP and Bcam1349 function in the same pathway, regulating production of the putative biofilm matrix exopolysaccharide through modulating the expression of one or more genes in the Bcam1330–Bcam1341 gene cluster. To test this hypothesis further, we used qRT-PCR to compare transcript levels of the exopolysaccharide genes *bcam1330*, *bcam1331,* and *bcam1332* in both the Bcam1349-overproducing wild type/pBcam1349 strain and the c-di-GMP-overproducing wild type/pYedQ strain. We found that overproduction of Bcam1349 from the pBcam1349 plasmid in the wild type resulted in a 30-fold increased level of the *bcam1330* transcript, and an 11-fold increased level of the *bcam1331* transcript compared with the vector control strain. The level of the *bcam1332* transcript did not change significantly in the Bcam1349-overproducing strain compared with the vector control strain (Fig. 9). Elevated levels of intracellular c-di-GMP in the wild-type/pYedQ strain resulted in a 50-fold increased level of the *bcam1330* transcript, a 28-fold increased level of the *bcam1331* transcript, and a 2.3-fold increased level of the *bcam1332* transcript compared with the vector control strain (Fig. 9).

### Determination of the transcriptional start site of the *bcam1330* and *bcam1331* genes

To determine the transcriptional start site (TSS) of the *bcam1330* and *bcam1331* genes, we used the 5′ RACE method. Sequence analysis of the fragments that were generated by nested PCR revealed that the TSS for *bcam1330* is nucleotide A located 88-bp upstream of the ORF, whereas the TSS for *bcam1331* is nucleotide A located 64-bp upstream of the ORF (Fig. S1). Furthermore, a putative ribosomal binding site, GGAA, was present 38-bp upstream of the *bcam1330* ORF and 33-bp upstream of the *bcam1331* ORF (Fig. S1).

### Bcam1349 binds to *bcam1330* and *bcam1331* promoter DNA and this binding is enhanced by the presence of c-di-GMP

**Figure 10:**
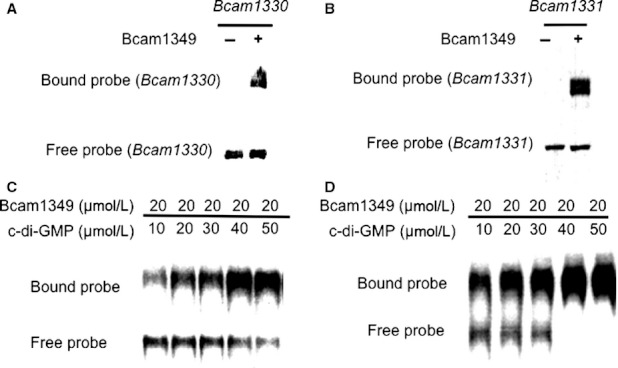
(A, B) Binding of Bcam1349 to the promoter regions of *bcam1330* and *bcam1331* genes assessed by the use of electrophoretic mobility shift assay (EMSA). Each lane contained 1.5 nmol/L Digoxigenin (DIG)-labeled Probe DNA, and in addition, the first lane contained 20 μmol/L purified GST-tag, whereas the following lanes contained 20 μmol/L of GST-tagged Bcam1349 protein. (C and D) EMSA assessment of the impact of cyclic diguanosine monophosphate (c-di-GMP) on Bcam1349 binding to the promoter regions of *bcam1330* and *bcam1331* genes. DIG-labeled promoter fragments were incubated with purified Bcam1349 protein in the presence of c-di-GMP as indicated.

We have previously shown that Bcam1349 binds to *bcs* promoter DNA to induce transcription (Fazli et al. 2011). The finding that the transcription of the *bcam1330* and *bcam1331* genes is increased by overexpression of Bcam1349 (Fig. 9) suggests that Bcam1349 may bind to the intergenic regions upstream of the *bcam1330* and *bcam1331* genes and activate their transcription. To test this hypothesis, an electrophoretic mobility shift assay (EMSA) was performed using Digoxigenin (DIG) labeled upstream regions of the *bcam1330* and *bcam1331* genes. The probes were combined with 20 μmol/L of C-terminally GST-tagged Bcam1349 protein and assessed for binding using EMSA. As a positive and a negative control, EMSA was performed in parallel using the *bcs* promoter region that was previously shown to bind Bcam1349 and the promoter region of the *otsA* (*bcam1573*) gene that does not bind to Bcam 1349 as probes, respectively (data not shown). As shown in Figure 10A and B, the probes for the *bcam1330* and *bcam1331* genes and the Bcam1349 protein formed a stable DNA-protein complex that migrated at a slower rate than unbound probes, whereas no band shift was observed with the negative control lacking the protein. In contrast, the Bcam1349 protein could not form a DNA-protein complex with the *otsA* promoter, indicating specificity in DNA binding (data not shown).

As Bcam1349 binds c-di-GMP in vitro (Fazli et al. 2011) and the transcription of *bcam1330* and *bcam1331* genes is increased in the presence of elevated intracellular levels of c-di-GMP, we surmised that the addition of c-di-GMP to the DNA-protein reaction mixtures would influence the binding of Bcam1349 to *bcam1330* and *bcam1331* promoter DNA. In accordance, the addition of c-di-GMP at concentrations in the μmol/L range resulted in an increase in the ability of Bcam1349 to bind promoter DNA as measured by retarded migration of the DNA-protein complex through the gel (Fig. 10C and D).

Taken together, the qRT-PCR and EMSA results are consistent with a model where Bcam1349 acts as an inducer of the transcription of the *bcam1330* and *bcam1331* genes, and c-di-GMP stimulates this transcriptional activity through binding to and promoting the activity of Bcam1349.

## Discussion

We recently provided evidence that the c-di-GMP signaling system is involved in the regulation of biofilm formation in *B. cenocepacia* and identified a c-di-GMP responsive transcriptional regulator, Bcam1349, which regulates expression of cellulose and fimbriae biosynthesis genes in a manner that is promoted by c-di-GMP (Fazli et al. 2011). Cellulose and aggregative fimbriae play important roles in biofilm formation by *S.* Typhimurium and *E. coli*, and their co-production results in formation of a rigid extracellular biofilm matrix (Zogaj et al. 2001; Römling 2002). However, our experiments with cellulose (*bcal1389*) and fimbriae (*bcal1677*) mutants suggested that cellulose and fimbriae are not essential for *B. cenocepacia* biofilm formation and stability under the conditions we tested. The mutants formed flow-cell biofilms that were quite similar to the wild type flow-cell biofilms, and the mutant biofilms were nearly as resistant to SDS treatment as the wild type biofilm.

Overproduction of the Bcam1349 protein in the wild type resulted in formation of wrinkled colonies on solid medium and thick pellicles at the air–liquid interface in static cultures, which are indicative of production of adhesive biofilm matrix components such as exopolysaccharides, adhesive proteins, and aggregative fimbriae (Rainey and Travisano 1998; Spiers et al. 2002, 2003; Friedman and Kolter 2004a,b; Branda et al. 2005). We initially thought that this observation might have been in part due to overproduction of cellulose and fimbriae in the pBcam1349-containing wild type strain, as the expression of cellulose and fimbriae biosynthesis genes were found to be downregulated in the *Bcam1349* mutant (Fazli et al. 2011). However, when we introduced the plasmid pBcam1349 into the cellulose and fimbriae mutants, the mutants formed wrinkled colonies on agar medium and thick pellicles in standing cultures similar to the pBcam1349-containing wild type, whereas the mutants without pBcam149 formed smooth colonies and did not form pellicles. Based on these results, we concluded that, although their expression is regulated by Bcam1349, cellulose and fimbriae are not important for *B. cenocepacia* biofilm formation and stability under the conditions tested. This also suggested that Bcam1349 regulates the expression of other genes encoding products that serve as the major components of the biofilm matrix and are possibly more important for mature biofilm formation and stability than cellulose and fimbriae. In an effort to identify the target genes affected by Bcam1349, we screened for genes required for the formation of wrinkled colonies in the pBcam1349-containing wild type strain and identified a putative exopolysaccharide gene cluster, Bcam1330–Bcam1341, required for wrinkled colony morphology, pellicle, and biofilm formation in *B. cenocepacia*.

The biosynthesis of exopolysaccharides can be divided into distinct steps, including formation of nucleotide sugar precursors, polymerization and transport across the inner membrane, and modification and transport from the periplasm to the extracellular medium (Franklin et al. 2011). Sequence analysis of the Bcam1330–Bcam1341 exopolysaccharide biosynthesis gene cluster revealed that it encodes proteins that have putative functions required for most of the above steps. The cluster appears to contain one gene, *bcam1340*, for sugar precursor production. It is predicted to encode a mannose-1-phosphate guanylyltransferase catalyzing the formation of GDP-mannose precursor. Currently, we do not know the sugar composition of the putative exopolysaccharide encoded by the Bcam1330–Bcam1341 gene cluster; however, if other nucleotide sugar precursors than GDP-mannose are required, then the synthesis of these nucleotide sugar precursors is probably derived from central carbon metabolism and uses enzymes from other polysaccharide synthesis pathways, similar to the situation in *P. aeruginosa* where AlgC is required for precursor synthesis for alginate, Psl, rhamnolipid, and LPS biosynthesis (Goldberg et al. 1993; Coyne et al. 1994; Olvera et al. 1999; Bryd et al. 2009; Ma et al. 2012). The Bcam1330–Bcam1341 cluster contains three genes predicted to encode glycosyltransferases, which may function in the assembly of polysaccharide repeating units. Two of the glycosyltransferases are predicted to be localized in the cytoplasm and the subcellular localization of the remaining one is unknown. Four proteins encoded in the cluster have inner membrane spanning domains and are therefore likely to be involved in the polymerization of the polysaccharide and transport from the inner membrane to the periplasm. One of the inner membrane proteins, Bcam1333, is predicted to encode an acyltransferase, which may be involved in the modification of the polysaccharide. One of the genes in the cluster, *bcam1334*, encodes a predicted hydrolase. This protein may have a similar function in exopolysaccharide biosynthesis as AlgL, PelA, and PslG from the alginate, Pel, and Psl polysaccharide biosynthesis machinery of *P. aeruginosa*, respectively, which are involved in quality control of the secreted polysaccharides (Franklin et al. 2011). The cluster encodes only one protein, Bcam1330, with an outer membrane spanning domain and a Poly_export domain (Pfam02563), probably involved in the export of the polysaccharide from the cell. It is a periplasmic protein and has a structural similarity to the *E. coli* outer membrane auxiliary lipoprotein Wza, which is responsible for the final stage of capsule polysaccharide export (Cuthbertson et al. 2009). The above-mentioned functions are annotated to the proteins based on their sequence similarity to other proteins with known function. However, a systematic analysis of the proteins encoded in the Bcam1330–Bcam1341 cluster is needed to determine their specific functions in biosynthesis of the putative exopolysaccharide.

Most *Bcc* bacteria have been shown to have the capacity to produce large amounts of the exopolysaccharide cepacian giving the bacteria a mucoid phenotype, and it is suggested that cepacian facilitates persistence of *Bcc* bacteria during lung infection in CF patients. Cunha et al. (2004) demonstrated that cepacian plays a role in biofilm maturation, in particular in the establishment of thick biofilms but is not necessary for initiation of biofilm formation. Nonmucoid *Bcc* strains have also been isolated from CF patients (Zlosnik et al. 2008), suggesting that not all *Bcc* bacteria are able to form cepacian. In fact, the commonly studied clinical isolates *B. cenocepacia* J2315 and K56-2 produce little or no cepacian, yet they are still able to form biofilms in vitro (Saldias et al. 2008; Zlosnik et al. 2008; McCarthy et al. 2010). *Burkholderia cenocepacia* J2315 is unable to produce cepacian due to a frameshift mutation in the glycosyltransferase encoding *bceB* gene of the cepacian biosynthesis gene cluster; however, its genome contains several other gene clusters associated with exopolysaccharide biosynthesis (Holden et al. 2009), one of which is the Bcam1330–Bcam1341 gene cluster described in this study. Similarly, *P. aeruginosa* produces at least three different exopolysaccharides that are involved at different stages of biofilm development (Ryder et al. 2007; Franklin et al. 2011). Alginate has been demonstrated as the major polysaccharide produced by the mucoid isolates of *P. aeruginosa*. However, nonmucoid *P. aeruginosa* strains, which are the first to colonize CF patients, were found not to produce alginate as the major exopolysaccharide, and recent studies have demonstrated that alginate is in fact not required for initiation of biofilm formation by these nonmucoid strains (Wozniak et al. 2003; Stapper et al. 2004). Instead, the nonmucoid *P. aeruginosa* strains were found to produce the polysaccharides Pel and Psl, which have differing roles with respect to adherence and biofilm formation depending on the strain. Studies demonstrated that the Pel polysaccharide is not essential for bacterial adherence to surfaces but involved in further maturation of biofilms, while the Psl polysaccharide is involved in bacterial adherence to surfaces and in the maintenance of already established biofilms (Friedman and Kolter 2004a,b; Jackson et al. 2004; Matsukawa and Greenberg 2004; Ma et al. 2009; Colvin et al. 2011). A similar situation may exist in *B. cenocepacia*, and accordingly, the presence of multiple gene clusters for exopolysaccharide biosynthesis suggests that the bacterium may require and produce different polysaccharides at different stages of biofilm formation, probably as a response to diverse environmental stimuli.

Overproduction of c-di-GMP and Bcam1349 in *B. cenocepacia* both resulted in the same phenotypic alterations such as wrinkled colony morphology on solid medium, thick pellicles in static liquid cultures, and increased biofilm formation in flow-cells; and mutations in multiple genes of the Bcam1330–Bcam1341 exopolysaccharide biosynthesis gene cluster reverted these phenotypes. The Bcam1349 protein was shown to bind to the promoter region of the *bcam1330* and *bcam1331* genes, and this binding was enhanced in the presence of c-di-GMP. Furthermore, overproduction of c-di-GMP or Bcam1349 resulted in elevated transcript levels of the *bcam1330* and *bcam1331* genes. We previously demonstrated that Bcam1349 plays a role downstream in c-di-GMP signaling by acting as a c-di-GMP responsive transcriptional regulator (Fazli et al. 2011). Based on our previous and current results, we propose a model where binding of c-di-GMP to Bcam1349 enhances the protein's affinity for the promoter DNA region of the *bcam1330* and *bcam1331* genes in the Bcam1330–Bcam1341 gene cluster and thereby activates exopolysaccharide production and promotes biofilm formation by the bacterium.

In contrast to other opportunistic pathogens, *Bcc* bacteria are not normally carried as commensal organisms, suggesting that the infection is either acquired in the hospital setting or from the environment (Hutchinson et al. 1996; Oie and Kamiya 1996). Most clinical isolates of *Bcc* bacteria display the mucoid phenotype, whereas the original environmental strains usually are nonmucoid. Other exopolysaccharides than cepacian may be more important for survival of the bacteria in the environment and may be required during initial colonization of the CF lung prior to switch to a mucoid phenotype. In addition, some *Bcc* strains, for example, *B. cenocepacia* J2315, are nonmucoid and do not produce cepacian, yet they survive in the CF lung despite aggressive antimicrobial therapy and host immune response, indicating that they may use other exopolysaccharides than cepacian as components of the biofilm matrix during biofilm formation in vivo. Our results in this study suggest that the product encoded by the Bcam1330–Bcam1341 cluster is a major exopolysaccharide that provides structural stability to biofilms formed by *B. cenocepacia*.

## Supporting Information

**Figure S1.** Mapping of the 5′ end of the *Bcam1330* and *Bcam1331* transcript by the 5′ RACE method.
