# Supplementary figures and images for: The exopolysaccharide gene cluster Bcam1330–Bcam1341 is involved in Burkholderia cenocepacia biofilm formation, and its expression is regulated by c-di-GMP and Bcam1349
Source: Microbiologyopen. 2012 Dec 25;2(1):105–22. doi: 10.1002/mbo3.61 (PMC3584217; doi:10.1002/mbo3.61)

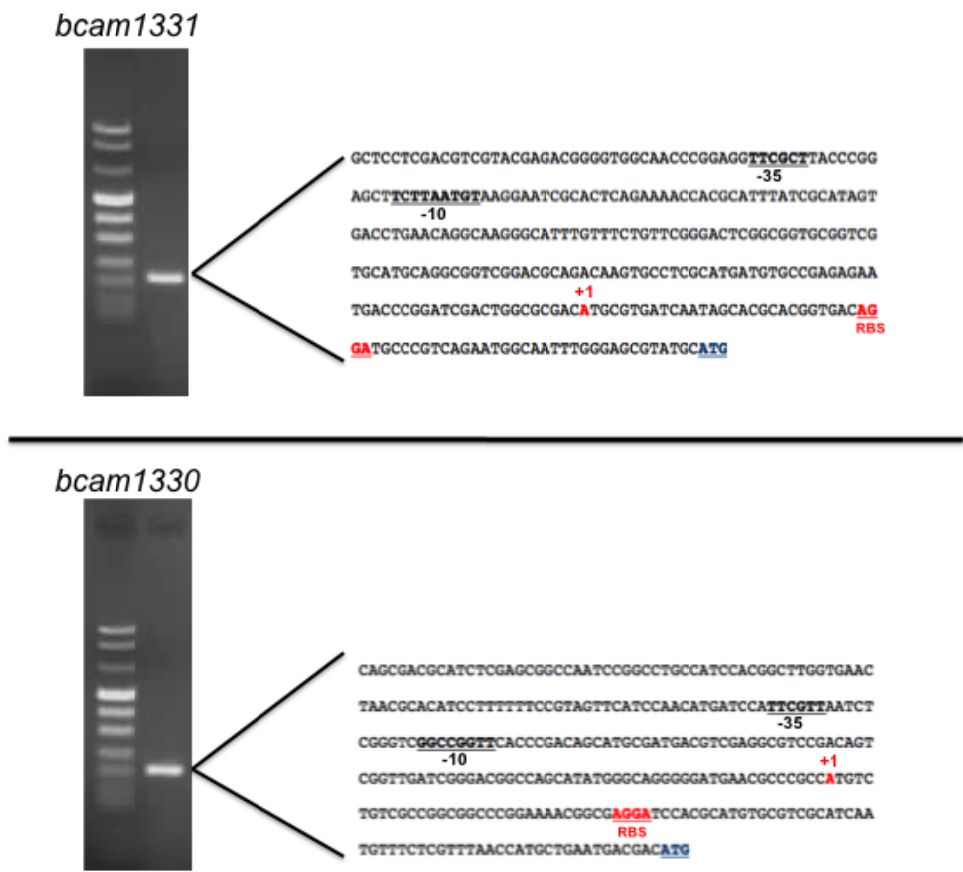

**Figure S1.** Mapping of the 5' end of the *Bcam1330* and *Bcam1331* transcript by the 5' RACE method.

Supplement: Supplementary file 1 [file mbo30002-0105-SD1.pdf]
